# A facile energy-saving route of fabricating thermoelectric Sb_2_Te_3_-Te nanocomposites and nanosized Te

**DOI:** 10.1098/rsos.180698

**Published:** 2018-10-17

**Authors:** En-Yu Liu, Fei-Hung Lin, Zong-Ren Yang, Chia-Jyi Liu

**Affiliations:** 1 National Changhua Senior High School, Changhua 500, Taiwan, Republic of China; 2 Department of Physics, National Changhua University of Education, Changhua 500, Taiwan, Republic of China

**Keywords:** thermoelectric composites, Sb_2_Te_3_, energy-saving synthesis, nanoparticles

## Abstract

A facile energy-saving route is developed for fabricating Sb_2_Te_3_-Te nanocomposites and nanosized Te powders. The fabrication route not only avoids using organic chemicals, but also keeps the energy consumption to a minimum. The fabrication procedure involves two steps. Energetic precursors of nanosized powders of Sb and Te are produced at room temperature followed by hot pressing at 400°C under 70 MPa for 1 h. The resulting Sb_2_Te_3_-Te nanocomposite exhibits enhanced power factor. The dimensionless figure of merit *zT* value of the Sb_2_Te_3_-Te nanocomposite is 0.29 at 475 K.

## 1. Introduction

**Figure 1.**
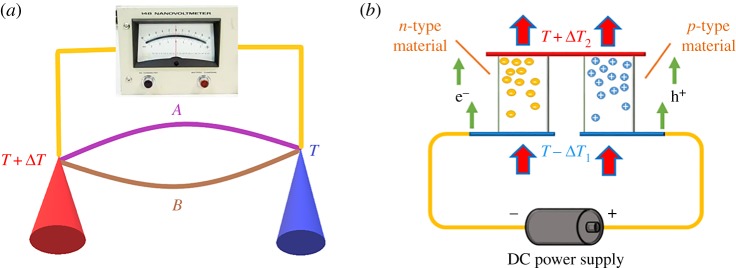
Thermoelectric circuits illustrating (*a*) the Seebeck effect and (*b*) the Peltier effect.

Figure 1 shows the thermoelectric circuit consisting of two junctions with each junction connected by two dissimilar conductors. Based on the thermoelectric phenomena, there are five effects operating simultaneously in the circuit, namely, the Seebeck, Peltier, Thomson, Joule and Fourier effects [1]. The Seebeck effect is an electromotive force (emf) generated in the circuit by the temperature gradient across two junctions (one hot junction and the other cold junction), which can be used to make thermoelectric generators (TEG). TEG can be used to recover waste thermal energy and supply electricity for electronic devices such as fans, mobile phones, lights, radios and microsensors in areas where electric power is not available. Besides, the solar thermoelectric generators using solar heat as heat source can be stored in energy storage materials [2–4]. The Peltier effect is the reverse thermoelectric phenomenon of the Seebeck effect. The Peltier effect is one junction in the circuit becoming colder with its heat transferred to the other junction becoming hotter when an electrical current flows through the circuit, which can be used to make solid-state thermoelectric coolers (TEC). TEC can be used in a wide range of applications such as food and beverage cooling, black box cooling, constant temperature controlling, dehumidifying, cold plates, on-board refrigeration, electronics packaging cooling and semiconductor wafer probes. The Thomson effect is the generation or absorption of heat for a single current-carrying conductor along its length in the presence of temperature gradient. The Joule effect is the Joule heat generated when an electrical current flows through a conductor. The Fourier effect is the conductive heat transfer between two junctions with temperature difference. Thermoelectric devices are based on these five effects acting in combination and involving interactions between the heat flow and electric charge flow through solid bodies. Thermoelectric materials are used to fabricate thermoelectric modules for thermal energy conversion to direct current electricity via the Seebeck effect and solid-state refrigerators via the Peltier effect.

Thermoelectric modules are made of *p*- and *n*-type thermoelectric materials. To evaluate whether a material is a good thermoelectric material candidate, dimensionless figure of merit *zT* is adopted as a criterion. The value of *zT* is expressed by

1.1 $$zT=\frac{\sigma S^{2}}{\kappa },$$

where *σ*, *S*, *κ* and *T* are the electrical conductivity, thermopower (or Seebeck coefficient), total thermal conductivity and absolute temperature, respectively. The total thermal conductivity *κ* consists of electronic thermal conductivity (*κ*_e_) and lattice thermal conductivity (*κ*_lattice_), that is, *κ* = *κ*_e_ + *κ*_lattice_. Therefore, a material with high electrical conductivity, large thermopower and low thermal conductivity would lead to high *zT* values. For TEG, the power generation efficiency *η*_TE_ is expressed as [1]

1.2 $$\eta_{\mathrm{TE}}=\left(\frac{T_{H}-T_{C}}{T_{H}}\right)\left(\frac{\sqrt{1+ZT_{M}}-1}{\sqrt{1+ZT_{M}}+(T_{C}/T_{H})}\right),$$

where *T*_M_ *=* (*T*_H_ *+ T*_C_)/2 is the mean temperature; *T*_H_ and *T*_C_ are the temperatures of heat source and heat sink, respectively. For TEC, the coefficient of performance (COP) *ϕ* is given as [1]

1.3 $$\phi =\;\frac{(S_{P}-S_{N})IT_{C}-K\Delta T-(1/2)I^{2}R}{I[(S_{P}-S_{N})\Delta T+IR]},$$

where *S_P_* and *S_N_* are the thermopower for *p*- and *n*-leg of the Peltier module; *I* is the current flowing through the module, *K* the total thermal conductance, *ΔT* = *T*_H_ – *T*_C_, and *R* the total electrical resistance.

Quite a few thermoelectric material systems with high dimensionless figure of merit *zT* have been discovered in the recent decade-long research efforts [5–17]. High *zT* values are required to have high thermoelectric performance. Unfortunately, high electrical conductivity is often accompanied by large thermopower and high electronic thermal conductivity. Hence, it is a common practice to optimize these transport parameters in the thermoelectric research through a number of strategies.

**Figure 2.**
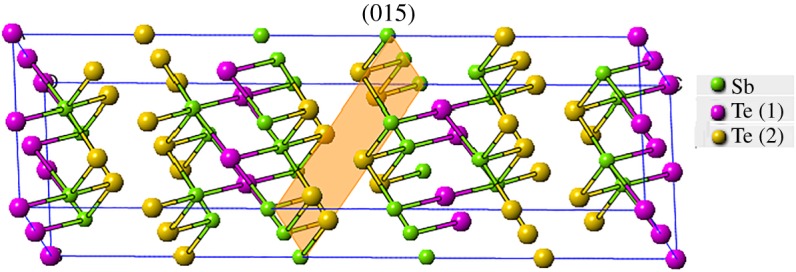
Crystal structures of Sb_2_Te_3_. The shaded area indicates the (015) plane, showing the largest peak intensity in the power X-ray diffraction pattern.

The crystal structure of Sb_2_Te_3_, as shown in figure 2, is composed of layers of identical atoms following the sequence of a quintet -Te(1)-Sb-Te(2)-Sb-Te(1)- stack along the *c* axis of the Sb_2_Te_3_ unit cell. The weak van der Waals bonding between Te(1)-Te(1) of two quintets has been attributed to their anisotropic transport properties. Sb_2_Te_3_ is one of the end members of commercial thermoelectric materials Bi_2−x_Sb_x_Te_3_. Besides, Sb_2_Te_3_ is a *p*-type three-dimensional topological insulator [18].

It is now well known that nanostructuring of a material could reduce *κ*_lattice_ significantly due to its high interface density and is therefore used as one of the strategies to enhance *zT* values. Nanostructured materials can be fabricated using solution methods of synthesizing nanosized powders in combination with appropriate consolidation techniques. Since the reactants have intimate mixing for reactions carried out using solution methods such as sol-gel, co-precipitation and hydrothermal methods, the final product can thus be obtained at relatively low temperatures. The low-energy input in turn provides an energy-saving and environmentally green means for fabricating nanostructured materials. There are several successful examples for producing high *zT* values of thermoelectric materials using solution methods [19–33].

Since using solid-state reactions or melting processes would require high-energy input and consume tremendous amount of energy, we aim to synthesize thermoelectric materials using fabrication routes with less energy consumption either in the synthesis and/or consolidation stage [34,35]. In this paper, we demonstrate a green energy-saving route for fabricating Sb_2_Te_3_-Te nanocomposites, which only requires hot pressing the constituent nanopowders at 400°C under an applied pressure of 70 MPa for 1 h.

## 2. Experiments

In a typical experiment, SbCl_3_ and commercial Te powders were weighed according to the desired molar ratio of Sb/Te. The Te powders were first put into 100 ml DI water inside a plastic beaker, followed by the addition of NaOH and sodium borohydride (NaBH_4_) as a mild reducing agent. Antimony chloride (SbCl_3_) was then added to the above alkaline reductive solution, which is left at room temperature for reaction to proceed. The resulting product was filtered and washed using deionized water, followed by drying in oven at 80°C for 12 h. The Sb_2_Te_3_-Te nanocomposite was fabricated by simply hot pressing the precursors at 400°C and an applied pressure of 70 MPa for 1 h.

The phase identification of as-synthesized powders was carried out using a Shimadzu XRD-6000 diffractometer equipped with Fe K*α* radiation. The scanning electron micrograph image was taken using a JEOL JSM-7610F field emission scanning electron microscope (FE-SEM). High-resolution transmission electron microscope (HRTEM) images were taken using a JEOL JEM-2100F transmission electron microscope. The composition of Sb_2_Te_3_-Te composites was analysed using energy dispersive spectroscopy (EDS) with Oxford MAX 50 detector. Electrical resistivity and thermopower were simultaneously measured using Setaram SeebeckPro measurement system. The precision of Seebeck coefficient and electrical resistivity is ±7% and ±10%, respectively. The Seebeck coefficient and electrical resistivity were measured using four-probe and steady-state techniques, respectively. Two type S thermocouples were used to measure the temperature difference between hot and cold ends of the sample. Hall carrier concentration and mobility were measured using Van der Pauw methods. Thermal conductivity measurements were carried out using transient plane source techniques with very small temperature perturbations of sample material by a hot disc thermal constants analyser. The uncertainty for the thermal conductivity is about ±5%. The electricity consumption was measured using a wattmeter which confirms to CNS 14607 (Chinese National Standards) with the uncertainty of ±0.3–0.4%. The electricity consumption of fabricating Sb_2_Te_3_-Te nanocomposites in this work is 2.8 kWh.

## 3. Results and discussion

**Figure 3.**
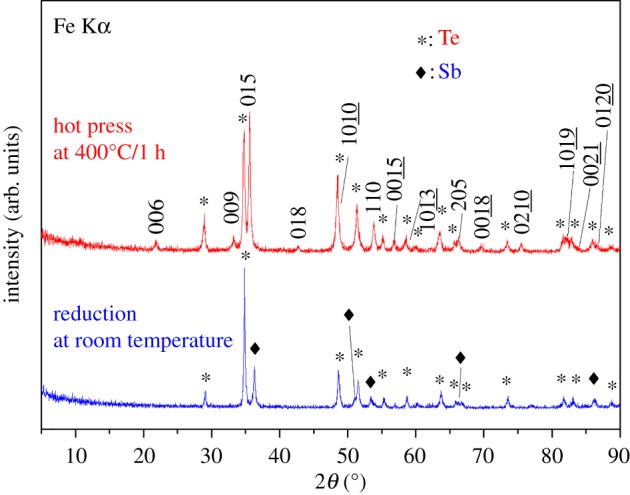
Powder X-ray diffraction patterns for room-temperature synthesized precursors and the Sb_2_Te_3_-Te nanocomposite obtained by hot pressing the precursors at 400°C and 70 MPa for 1 h. The refection peaks labelled with Miller indexes arise from Sb_2_Te_3_.

**Figure 4.**
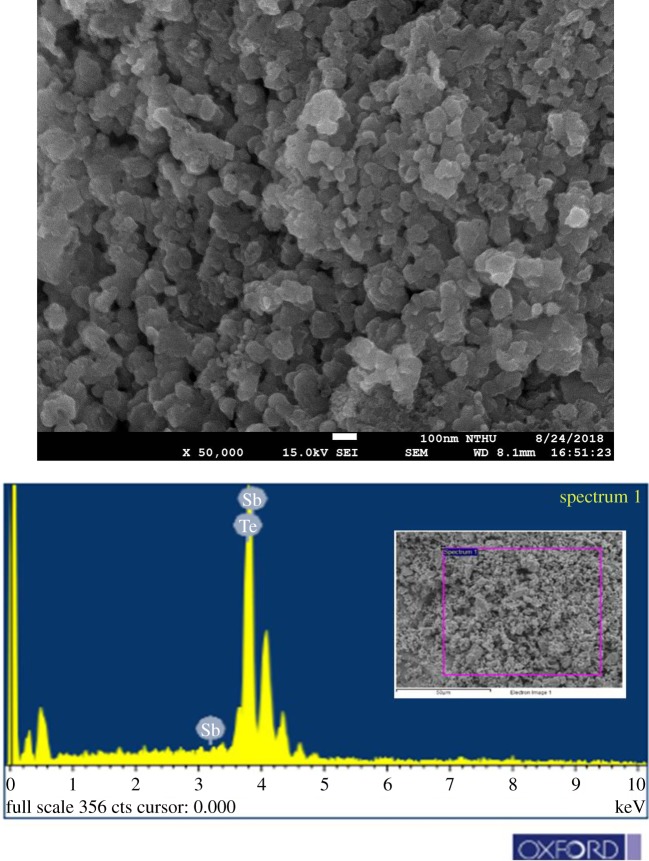
Field emission scanning electron micrograph and EDS analysis of the Sb_2_Te_3_-Te nanocomposite.

Figure 3 shows the powder X-ray diffraction patterns (XRD) of as-synthesized precursor and the Sb_2_Te_3_-Te nanocomposites. The as-synthesized precursor consists of Sb and Te as shown in figure 3. Reflection peaks corresponding to the rhombic Sb_2_Te_3_ are labelled by Miller indexes. The precursor transforms to Sb_2_Te_3_-Te nanocomposites using hot press at the condition of 70 MPa and 400°C for 1 h. Figure 4 shows the FE-SEM images and the EDS analysis of the Sb_2_Te_3_-Te nanocomposites. It clearly indicates as-synthesized precursors are nanopowders with various sizes. The characteristic X-ray L*α* line has the energy of 3.604 and 3.769 keV for Sb and Te, respectively. The difference between them is only 165 eV which almost reaches the energy resolution of the detector. Therefore, the obtained atomic percentage of Sn and Te is not quantitatively accurate and the analysis can only be considered qualitative results. However, the analysed results indicate that the composition of the nanocomposite is Te-rich, which is consistent with the coexistence of Sb_2_Te_3_ and Te phases in the XRD experiments.

**Figure 5.**
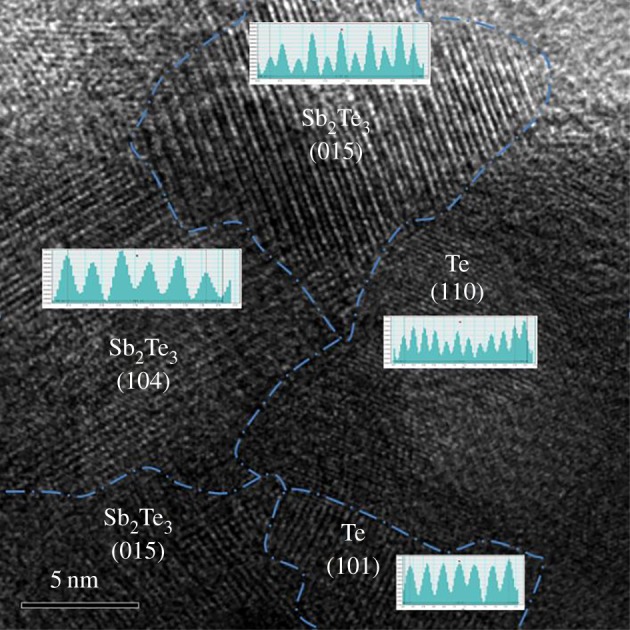
High-resolution transmission electron micrograph of Sb_2_Te_3_-Te nanocomposite.

Figure 5 shows the HRTEM image of as-fabricated Sb_2_Te_3_-Te nanocomposites. We estimate the spacing for each grain and find that they correspond to lattice planes of (015) and (104) for Sb_2_Te_3_ and (110) and (101) for Te, respectively. These results clearly indicate the formation of nanocomposite.

Based on the above results, the formation mechanism of Sb_2_Te_3_-Te nanocomposite is proposed to occur via the following steps:

3.1 $$3\mathrm{Te}+6OH^{-}⇄\;2Te^{2-}+{\mathrm{TeO}}_{3}^{2-}\;+3H_{2}O,$$

3.2 $${\mathrm{TeO}}_{3}^{2-}+{\mathrm{BH}}_{4}^{-}⇌{\mathrm{Te}}_{x}^{2-}+H_{2}{\mathrm{BO}}_{3}^{-}+H_{2},$$

3.3 $${\mathrm{Te}}_{x}^{2-}\to (x-1)\mathrm{Te}+Te^{2-},$$

3.4 $$4\mathrm{SbC}l_{3}\;+\;3\mathrm{NaB}H_{4}\to 4\mathrm{Sb}H_{3}+3\mathrm{NaCl}+3\mathrm{BC}l_{3},$$

3.5 $$2\mathrm{Sb}H_{3}\to 3H_{2}+2\mathrm{Sb}$$

3.6 $$\;\mathrm{and}\;2\mathrm{Sb}\;+3\mathrm{Te}\overset{\Delta }{⟶}Sb_{2}Te_{3}.$$

**Figure 6.**
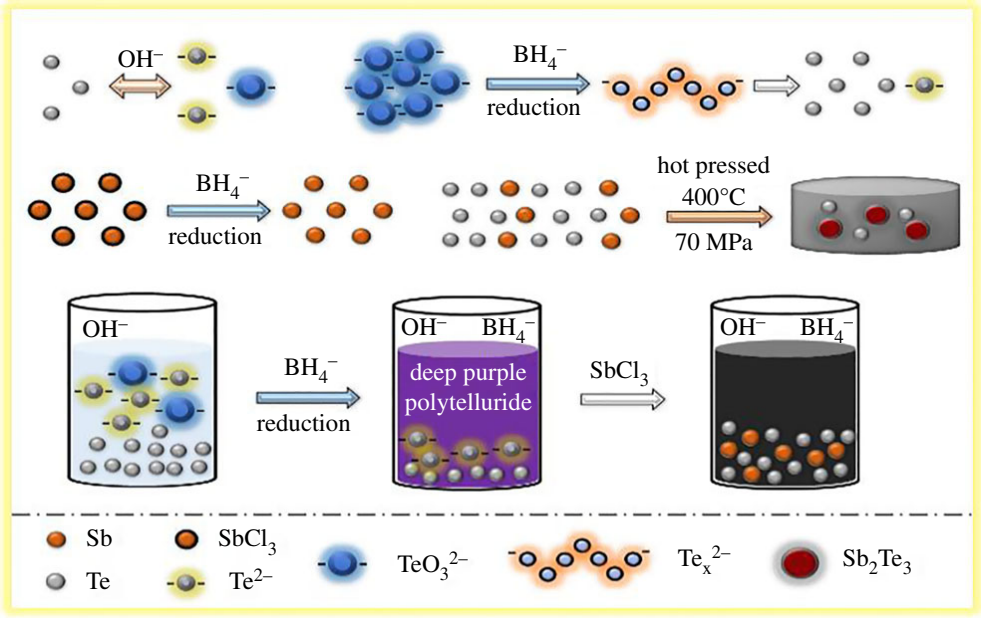
Illustration of growth mechanism for the Sb_2_Te_3_-Te nanocomposite synthesized at room temperature, followed by hot pressing at 400°C under 70 MPa for 1 h.

As shown in equation (3.1), tellurium dissolves in alkaline solution and undergoes a disproportionation reaction to give Te^2−^ and TeO_3_^2−^. Upon addition of NaBH_4_, TeO_3_^2−^ is reduced by ${\mathrm{BH}}_{4}^{-}$ to polytellurides ${\mathrm{Te}}_{x}^{2-}$ with evolution of H_2_ gas [34–36]. The presence of polytellurides is evidenced by its deep purple colour. The polytellurides ${\mathrm{Te}}_{x}^{2-}$ then undergo another disproportionation reaction to give Te and Te^2−^ as seen in equations (3.2) and (3.3). The subsequent addition of SbCl_3_ to the purple solution consisting of polytellurides gives stibine SbH_3_, which quickly converts to elemental antimony upon the action of thermal energy arising from the exothermal reaction in the process. The resulting black precursors of Sb and Te can be understood in equations (3.1)–(3.5). Unfortunately, the precursors of elemental Sb and Te cannot react at room temperature to form Sb_2_Te_3_. Nevertheless, the Sb_2_Te_3_-Te nanocomposite is obtained by simply hot pressing the precursors at 400°C and 70 MPa for 1 h. One of the interesting findings is the reversible reaction in equation (3.2). Figure 6 illustrates the mechanism of the formation of Sb_2_Te_3_-Te nanocomposite.

**Figure 7.**
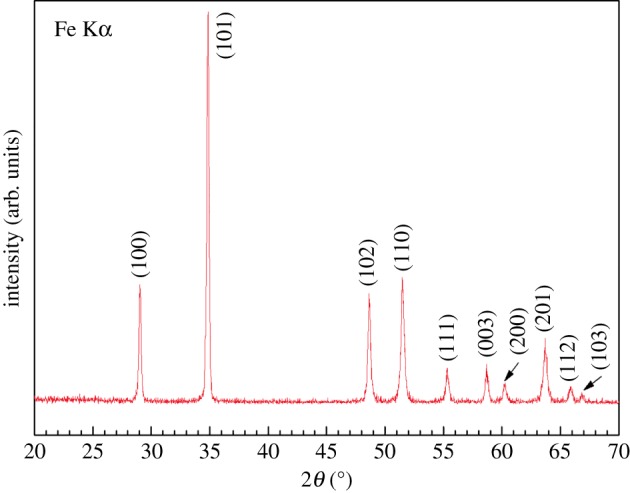
Powder X-ray diffraction patterns of as-synthesized Te nanopowders.

**Figure 8.**
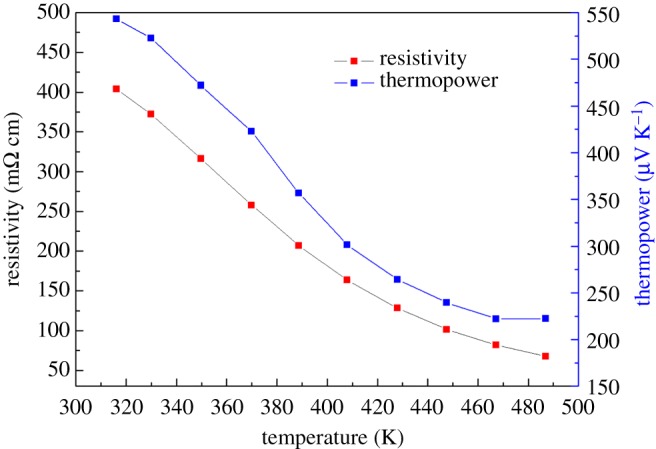
Temperature dependence of electrical resistivity and thermopower for the compacted nanosized Te bulk obtained at room temperature followed by sintering in an evacuated Pyrex ampoule at 400°C for 10 h.

In order to understand the effects of Te on the electronic transport of Sb_2_Te_3_-Te nanocomposites. We adopt the same procedure for fabricating nanostructured Te without adding SbCl_3_ and measure its electronic transport as a function of temperature. Figure 7 shows the XRD patterns of the room temperature synthesized Te nanopowders, which clearly indicates the peak broadening of the Te nanopowders as evidenced by an increase of the full width at half maximum (FWHM) when compared with the commercial powders. Using Sherrer's formula for X-ray particle size determination [37], the as-synthesized nanopowders of Te are estimated to have an average grain size of 38 nm. Figure 8 shows the temperature dependence of electrical resistivity and thermopower for the compacted Te synthesized at room temperature followed by sintering at 400°C for 10 h in an evacuated ampoule. The electrical resistivity and thermopower at 316 K is approximately 404 mΩ cm and 543 µV K^−1^. Both the resistivity and thermopower decrease with increasing temperature, indicating non-metal-like transport behaviour.

**Figure 9.**
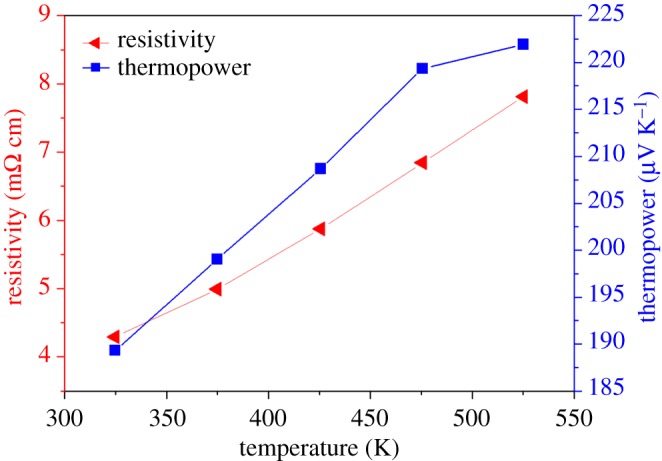
Temperature dependence of electrical resistivity and thermopower for the Sb_2_Te_3_-Te nanocomposite.

**Figure 10.**
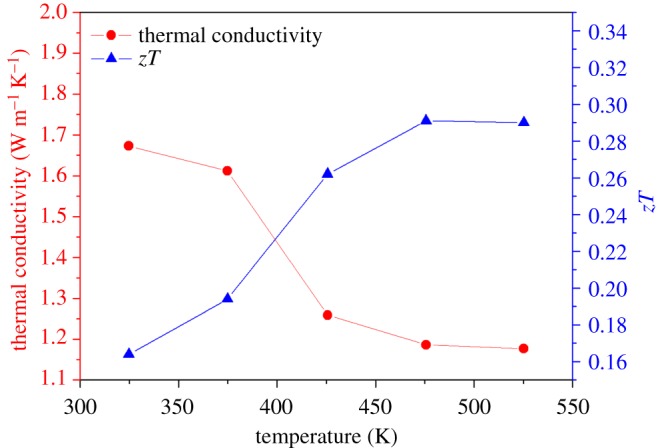
Temperature dependence of thermal conductivity and *zT* for the Sb_2_Te_3_-Te nanocomposite.

Figure 9 shows the temperature dependence of electrical resistivity and thermopower of the Sb_2_Te_3_-Te nanocomposite synthesized at room temperature followed by hot pressing at 400°C and 70 MPa for 1 h. The electrical resistivity and thermopower at 325 K is 4.29 mΩ cm and 189.3 µV K^−1^, respectively. Both the electrical resistivity and thermopower of the Sb_2_Te_3_-Te nanocomposite increase with increasing temperature. It is noted that the correlation of the electrical resistivity and thermopower follows the general trend, that is, a lower resistivity is usually accompanied by a smaller absolute thermopower. Hall measurements indicate the carrier concentration and mobility of the Sb_2_Te_3_-Te nanocomposite have room-temperature values of 7.6 × 10^18^ cm^−3^ and 111 cm^2^ V^−1^ s^−1^, respectively. Moreover, as compared to the literature data [38,39], the thermopower of our Sb_2_Te_3_-Te nanocomposite is remarkably large, which might arise from the energy filtering effects of Te in the nanocomposites. As a result, the power factor of the nanocomposite is significantly enhanced. The magnitude of the power factor is 8.4 µW cm^−1^ K^−2^ at 325 K. The nanostructured Te apparently plays a significant role in increasing the electrical resistivity and thermopower of the Sb_2_Te_3_-Te nanocomposite in light of its high resistivity and thermopower. Figure 10 shows the temperature dependence of *zT* for the Sb_2_Te_3_-Te nanocomposite synthesized at room temperature followed by hot pressing at 400°C and 70 MPa for 1 h. The thermal conductivity of the Sb_2_Te_3_-Te nanocomposite is 1.67 and 1.18 W m^−1^ K^−1^ at 325 K and 475 K, respectively. As a result, the *zT* is 0.29 at 475 K.

Single parabolic band (SPB) model can be used to analyse the thermoelectric transport by using an acoustic phonon scattering assumption [15,40,41]. The experimentally determined thermopower at a given temperature is first adopted to calculate the reduced Fermi level using the following equation:

3.7 $$S=\pm \frac{k_{B}}{e}\left[\frac{2F_{1}(\eta )}{F_{0}(\eta )}-\eta \right],$$

where $\eta =E_{F}/k_{B}T$ is the reduced Fermi energy, *k*_B_ the Boltzmann constant, *e* the elementary charge, *F_j_* (*η*) the Fermi integral, which is given by

3.8 $$F_{j}(\eta )=\int_{0}^{\infty }\frac{\xi^{j}}{1+e^{\left(\xi -\eta \right)}}d\xi .$$

The density of states effective mass of hole in the valence band is then calculated using experimentally determined Hall concentration, *n*_H_, and the following equations

3.9 $$m^{*}=\frac{h^{2}}{2k_{B}T}{\left[\frac{n_{H}\times r_{H}}{4\pi F_{1/2}(\eta )}\right]}^{2/3}$$

and

3.10 $$r_{H}=\frac{1.5F_{1/2}(\eta )F_{-1/2}(\eta )}{2F_{0}^{2}(\eta )},$$

where *h* is the Plank constant and *r*_H_ the Hall factor. Assuming identical thermal and electrical relaxations, we can estimate the electronic thermal conductivity, *κ_e_* = *LσT* using the Wiedemann–Franz relationship, where *L* is the Lorenz number, *σ* the electrical conductivity, and *T* the absolute temperature. The Lorenz number can be obtained using the following equation [42]:

3.11 $$L={\left(\frac{k_{B}}{e}\right)}^{2}\left[\frac{3F_{0}(\eta )F_{2}(\eta )-4F_{1}^{2}(\eta )}{F_{0}^{2}(\eta )}\right].$$

Despite the multi-band structure of Sb_2_Te_3_, we can use the above equations to roughly estimate some transport parameters for the Sb_2_Te_3_-Te nanocomposite at 325 K where the influence of minority carrier would be insignificant. As a result, we obtain the following material parameters at 325 K: reduced Fermi energy *η* = 0.3, density of states effective mass *m/m*_0_ = 0.46 (*m*_0_ = free electron mass), *L* = 1.642 × 10^−8^ W Ω K^−2^, and lattice thermal conductivity *κ*_lattice_ = 1.54 W m^−1^ K^−1^.

## 4. Conclusion

We have fabricated Sb_2_Te_3_-Te nanocomposites using a green energy-saving route with the synthesis carried out at room temperature followed by hot pressing at 400°C and 70 MPa for 1 h. The fabrication route not only avoids the use of organic substance but also keeps energy consumption to a minimum. The electricity consumption of fabricating Sb_2_Te_3_-Te nanocomposites in this work is 2.8 kWh.

## Supplementary Material

Powder x-ray diffraction, electronic, and thermal transport data for Sb2Te3-Te composite and Te nanostructured compact

## Data accessibility

The datasets, which can be obtained by double clicking the figures in ‘datasets_figure 2, 6, 7, 8, and 9_ESM.docx’ file with OriginPro software, are presented within this article and electronic supplementary material.
