# Supplementary material for: A facile energy-saving route of fabricating thermoelectric Sb2Te3-Te nanocomposites and nanosized Te
Source: R Soc Open Sci. 2018 Oct 17;5(10):180698. doi: 10.1098/rsos.180698 (PMC6227969; doi:10.1098/rsos.180698)
Supplement: Powder x-ray diffraction, electronic, and thermal transport data for Sb2Te3-Te composite and Te nanostructured compact [file rsos180698supp1.docx]

A facile energy-saving route of fabricating thermoelectric Sb_2_Te_3_-Te nanocomposites and nanosized Te

En-Yu Liu,^1^ Fei-Hung Lin,^2^ Zong-Ren Yang,^2^ and Chia-Jyi Liu*^,2^

^1^National Changhua Senior High School, Changhua 500, Taiwan

^2^Department of Physics, National Changhua University of Education, Changhua 500, Taiwan

Datasets can be obtained by double clicking the following figures with OriginPro software.

Figure S1. Powder x-ray diffraction patterns for (a) Sb_2_Te_3_ nanocomposites obtained by hot pressing Sb-Te precursors at 400°C and 70 MPa for 1 h. (b) Sb-Te precursors obtained using chemical reduction at room temperature. The refection peaks with Miller indexes arise from Sb_2_Te_3_. (Figure 2 in the text)

Figure S2. Power x-ray diffraction patterns of as-synthesized Te nanopowders. (Figure 6 in the text)

Figure S3. Temperature dependence of electrical resistivity and thermopower for the compacted Te bulk synthesized at room temperature followed by sintering at 400°C for 10 h. (Figure 7 in the text)

Figure S4. Temperature dependence of electrical resistivity and thermopower for the Sb_2_Te_3_-Te composites synthesized at room temperature followed by hot pressing Sb-Te precursors at 400°C under an applied pressure of 70 MPa for 1 h. (Figure 8 in the text)

Figure S5. Temperature dependence of zT for the Sb_2_Te_3_-Te composites synthesized at room temperature followed by hot pressing Sb-Te precursors at 400°C under an applied pressure of 70 MPa for 1 h. (Figure 9 in the text)
